# Application of psychosocial methods in work with an acutely psychotic patient: case report

**DOI:** 10.1192/j.eurpsy.2026.11699

**Published:** 2026-07-31

**Authors:** J. Lončar, T. Mastelić, J. Merćep, M. Ivandić, M. Galetić Pavlov, T. Glavina

**Affiliations:** Department of Psychiatry, University Hospital of Split, Split, Croatia

## Abstract

**Introduction:**

Psychosocial methods represent an important aspect of psychiatric care. Acute psychiatric patients, especially psychotic ones, are not always fully capable of such work. It usually takes some time for the psychological state to stabilize through the action of psychopharmacotherapy. Then patients can begin their journey of recovery, in which psychosocial methods play a significant role.

**Objectives:**

To present the case of a person who had an acute and transient psychotic disorder and in whom psychosocial methods were important in the treatment process.

**Methods:**

Review of medical records.

**Results:**

A patient in his early 30s is admitted to the Psychiatric Clinic with an acute psychotic disorder. He arrives accompanied by the police and is extremely agitated, aggressive, and threatens the staff. Over the course of three weeks, he gradually becomes more adequate and increasingly participates in psychosocial activities in the ward. He becomes involved in the work of the therapeutic community, raises the issue of a new television because the old one broke down not long before, and begins to maintain his bed, room, and personal hygiene. He suggests the topic of marijuana for psychoeducation, shares his experiences in the group, connects the harmful effects of marijuana with his current condition, gives a motivating speech about his recovery, but also suggests this to other members of the group. He comes forward and prepares a presentation about his travels. He prepares a PowerPoint presentation with pictures from various trips and hikes and presents it to other members of the group on the new television that he advocated for. He presents motivating plans for future trips and connects the symbolism of travel and hiking with the process of psychiatric treatment and recovery.

**Conclusions:**

In the presented case, we can see an acute psychiatric patient with psychotic symptoms who achieved great and visible progress, motivating him and others, in a short time. Psychosocial methods served as a driver and indicator of recovery. All this is important for the patient’s cooperation, which is usually a problem in psychiatry, and also for the success of his treatment. Although the application of psychosocial methods with acute psychiatric patients may seem discouraging at first, in the long term, it has an important place in treatment and yields significant results.

**Disclosure of Interest:**

None Declared

